# Supplementary figures and images for: Modelling the mechanics of exploration in larval Drosophila
Source: PLoS Comput Biol. 2019 Jul 5;15(7):e1006635. doi: 10.1371/journal.pcbi.1006635 (PMC6636753; doi:10.1371/journal.pcbi.1006635)

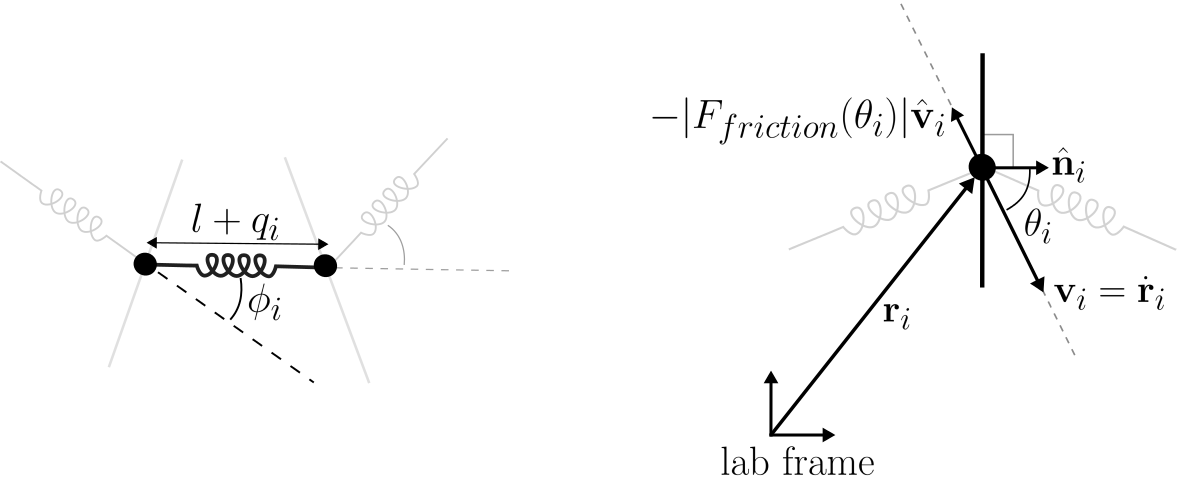

Supplement: S1 Fig — Internal coordinate system used to describe deformations of the larval body (left), and quantities used to describe substrate interaction (right). The friction force Ffriction acting on the i’th segment boundary is directed opposite to that boundary’s velocity vector vi, and has a magnitude which depends only upon the direction θi of the velocity vector relative to a unit vector n^i aligned with the local body axis (see text). Note that v^i=vi/∥vi∥ denotes a unit vector aligned with the boundary’s velocity vector. (TIF) [file pcbi.1006635.s001.tif]

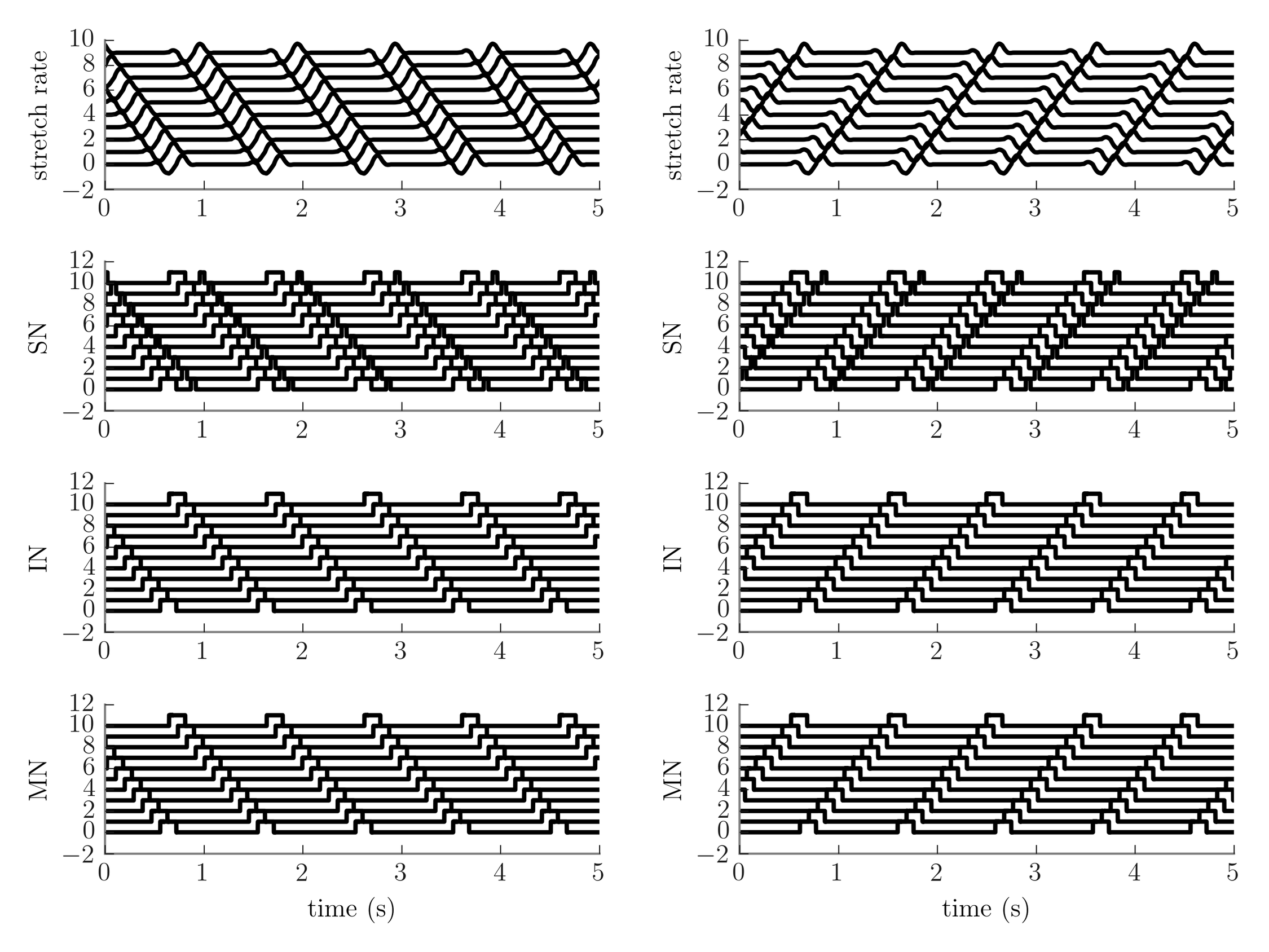

Supplement: S2 Fig — (from top to bottom) stretch rate, sensory neuron, interneuron, and motor neuron activation during forwards (left) and backwards (right) peristalsis. (TIF) [file pcbi.1006635.s002.tif]

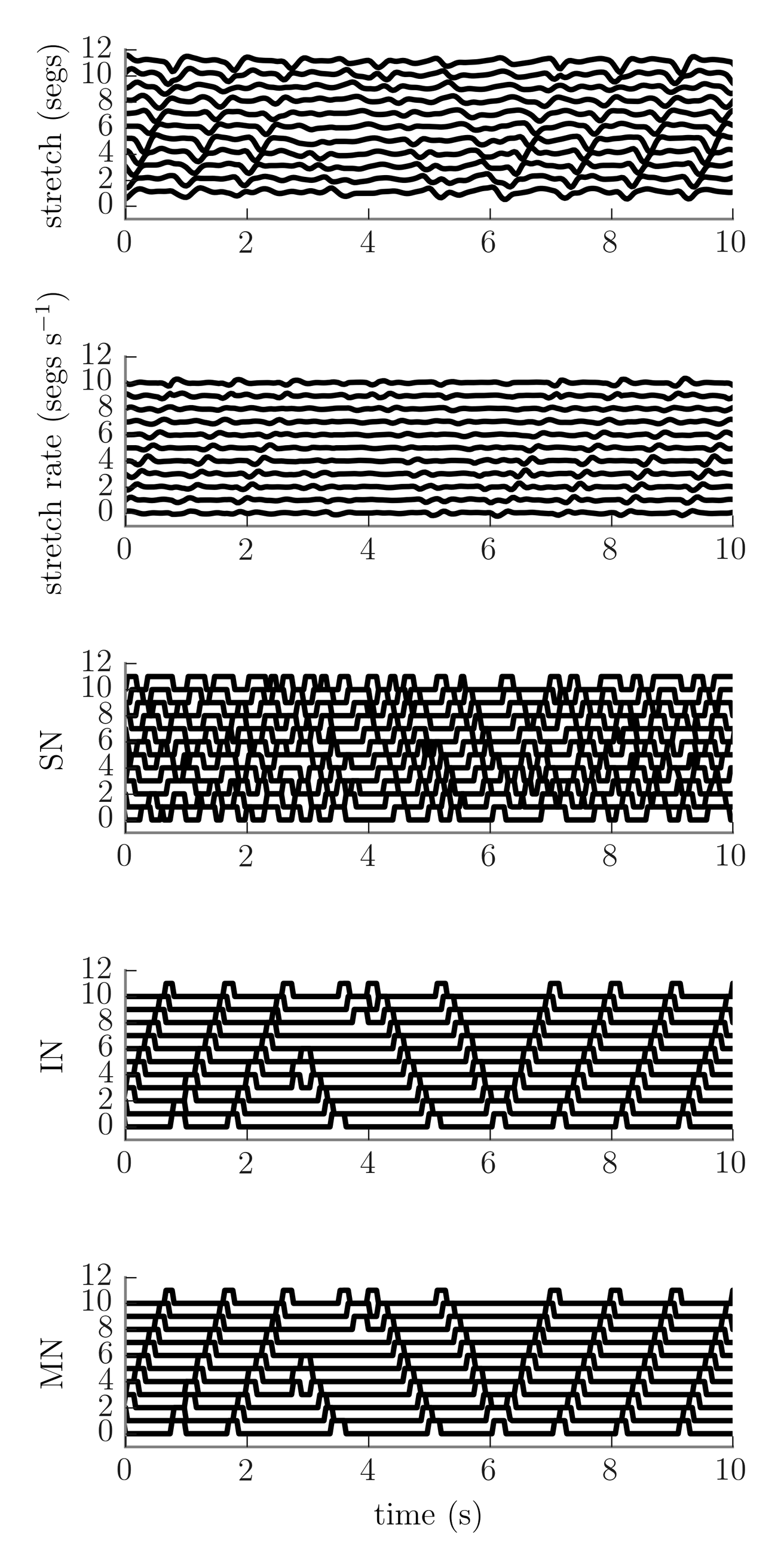

Supplement: S3 Fig — (from top to bottom) stretch, stretch rate, sensory neuron, interneuron, and motor neuron activation during planar motion. (TIF) [file pcbi.1006635.s003.tif]

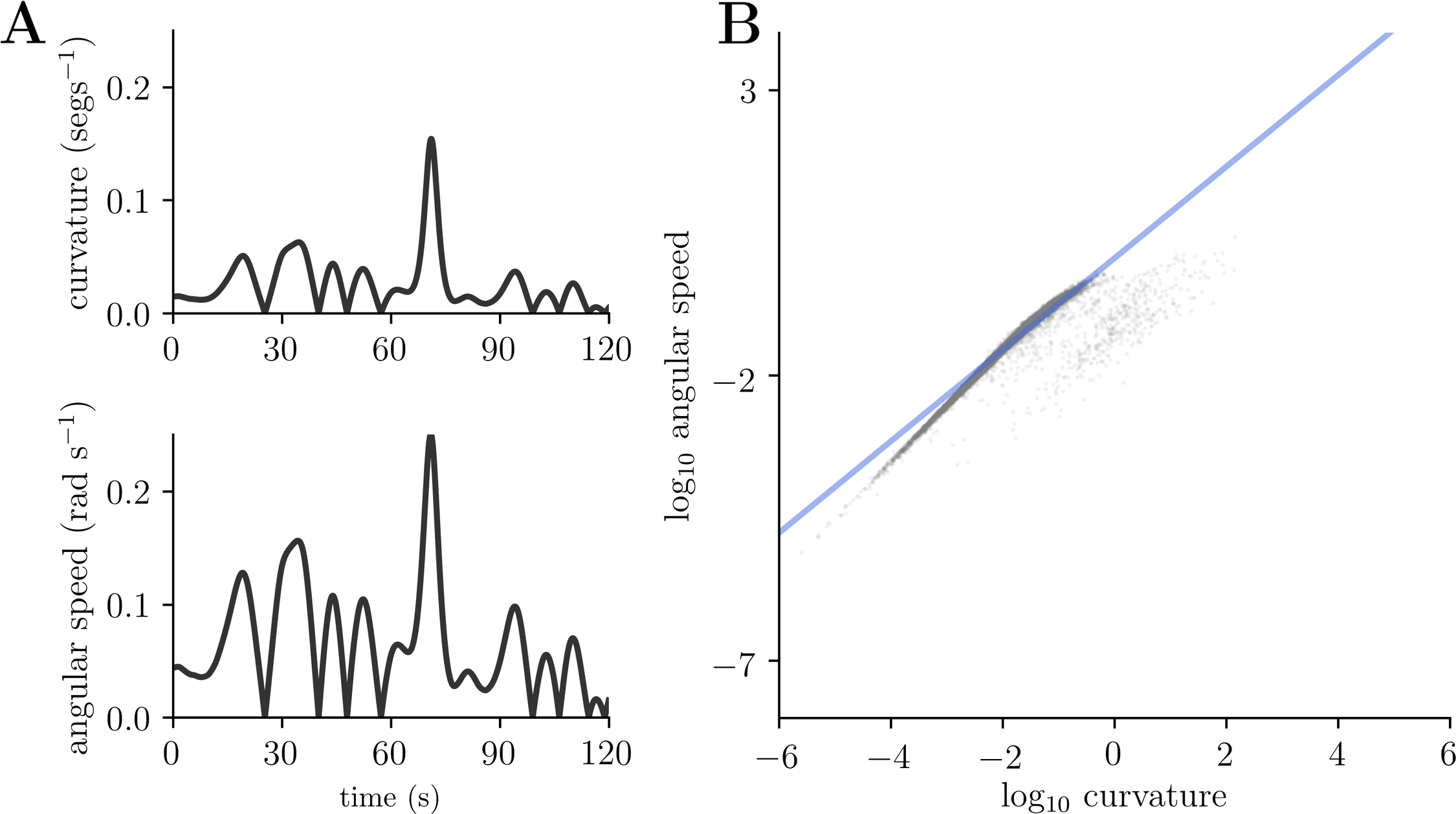

Supplement: S4 Fig — A: representative time-series of angular velocity and curvature. B: model data from all trials (grey points) compared to fit by a power law with scaling exponent β ≈ 0.8 (blue line, r2 ≈ 0.94). (TIF) [file pcbi.1006635.s004.tif]

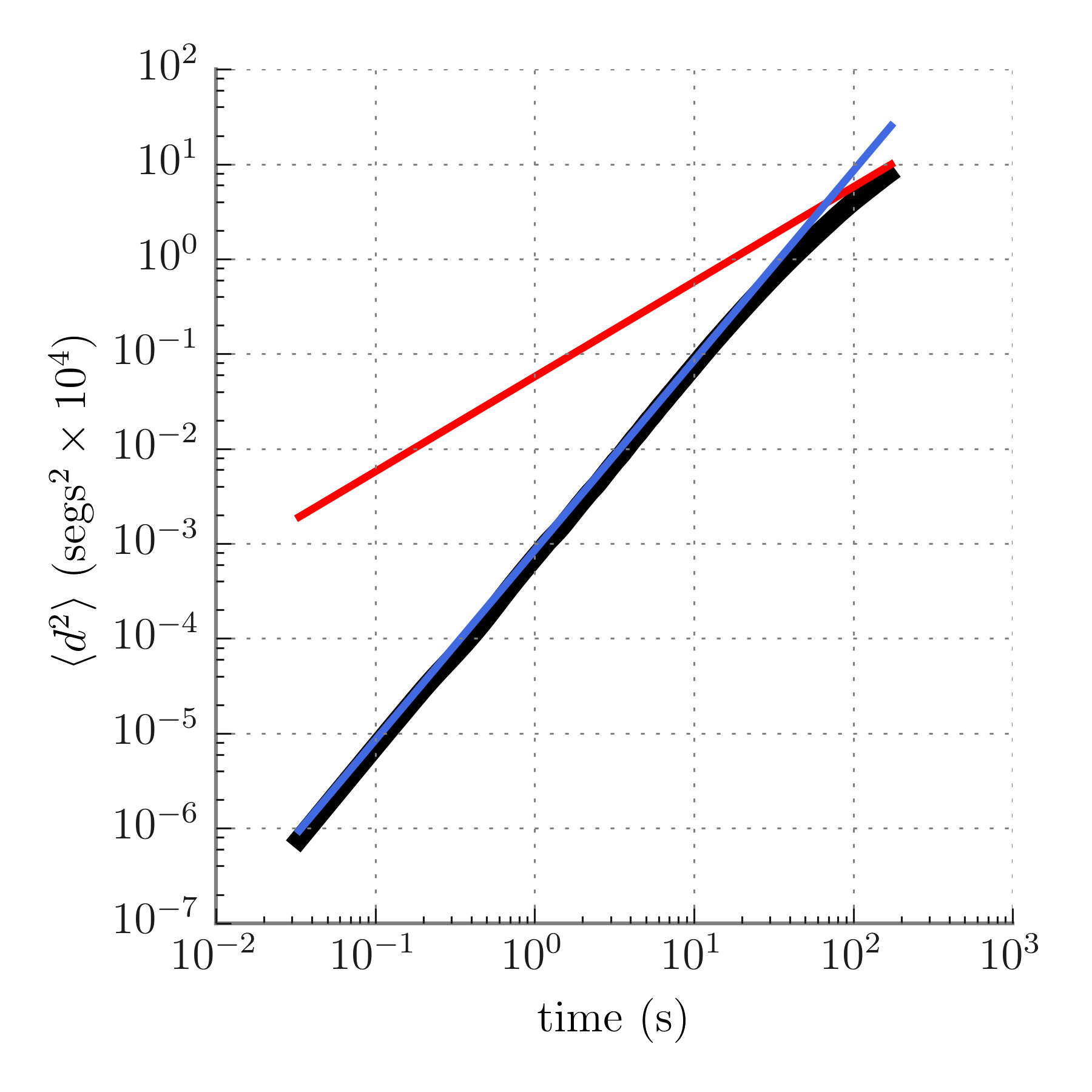

Supplement: S5 Fig — Initial quadratic growth (blue line, slope = 2) leads to asymptotic linear growth (red line, slope = 1). (TIF) [file pcbi.1006635.s005.tif]

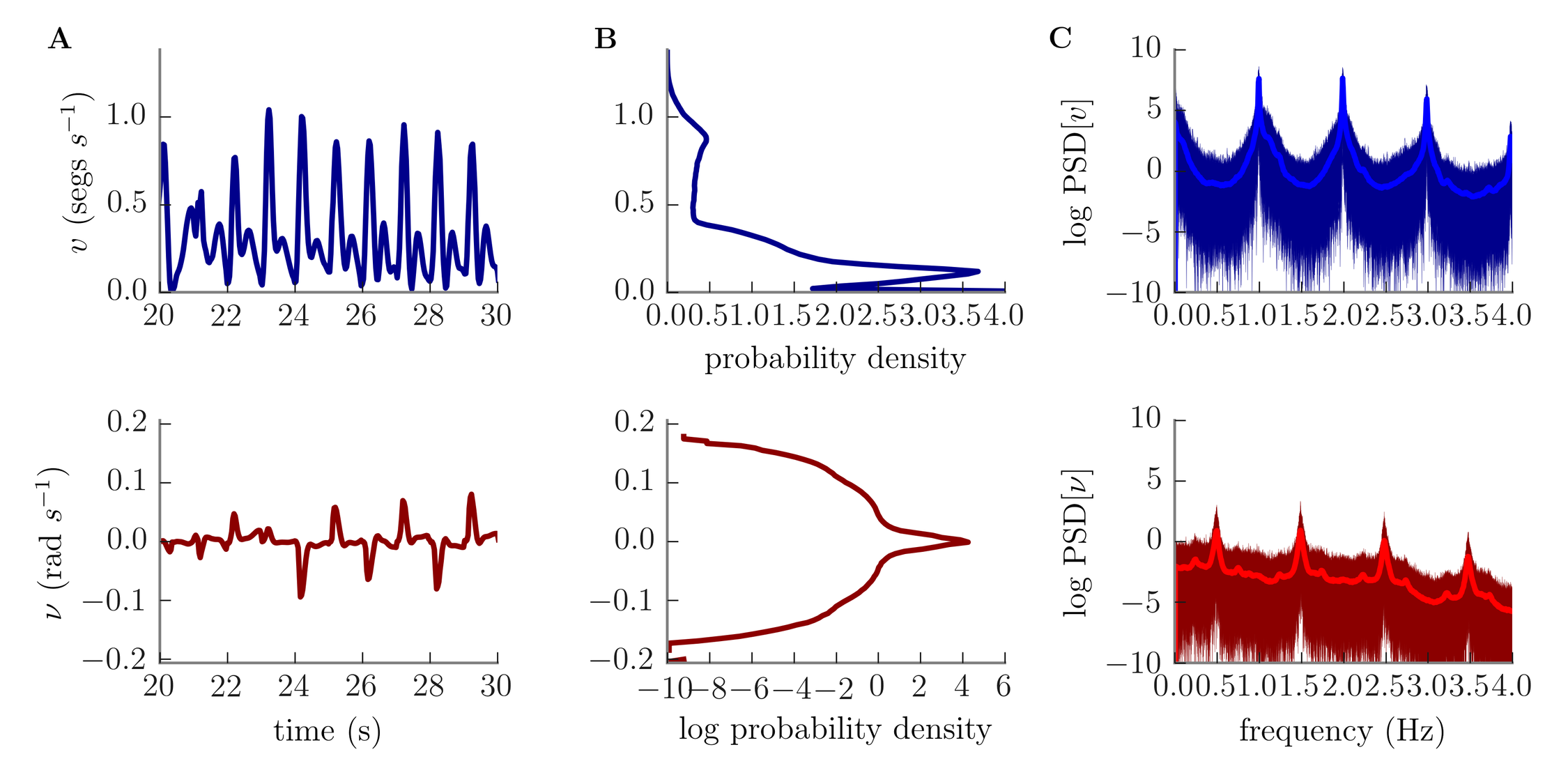

Supplement: S6 Fig — A: representative time series for v and ν. B: probability density of v and ν across all 1000 trials. C: individual (faint) and mean (bold) power spectra of v and ν. (TIF) [file pcbi.1006635.s006.tif]
