# Supplementary material for: Modelling the mechanics of exploration in larval Drosophila
Source: PLoS Comput Biol. 2019 Jul 5;15(7):e1006635. doi: 10.1371/journal.pcbi.1006635 (PMC6636753; doi:10.1371/journal.pcbi.1006635)
Supplement: S1 Appendix — (PDF) [file pcbi.1006635.s010.pdf]

S1 Appendix – Detailed model specification

In this appendix we provide a mathematical specification of our neuromechanical model. We will describe our mechanical model using the framework of Hamiltonian mechanics, since this is the natural setting for discussions of deterministic chaos within classical mechanics. To specify our model, we provide descriptions of the mechanical energy stored in the body and the power flow through the body. First, the total kinetic energy of the body is given by

$$T = \frac{1}{2} \sum_{i=1}^{N} m_i \dot{\mathbf{r}}_i^2 \tag{1}$$

where m_i is the mass of the i'th segment boundary and $\dot{\mathbf{r}}_i$ is its velocity measured relative to the substrate.

We assume that the cuticle stores elastic potential energy in both axial compression/expansion and in transverse bending. We further assume that there exists some equilibrium state where the potential energy is at a minimum, and at which point the length of the i'th body segment (bounded by masses m_i and m_{i+1}) is l_i. Axial deformation can then be conveniently described by a set of *axial stretches*, which measure the difference between each segment's current length and its equilibrium length

$$q_i = \|\mathbf{r}_{i+1} - \mathbf{r}_i\| - l_i, \quad i \in [1, N-1] \tag{2}$$

where the double bars indicate the standard Euclidean norm. We assume that the transverse potential energy is at a minimum when the masses are arranged in a straight line (i.e. when the midline is not curved). In this case it is convenient to measure transverse deformation by the *bending angle* made between successive body segments

$$\phi_i = \cos^{-1} \frac{\left[\mathbf{r}_i - \mathbf{r}_{i-1}\right]^T \left[\mathbf{r}_{i+1} - \mathbf{r}_i\right]}{\|\mathbf{r}_i - \mathbf{r}_{i-1}\| \|\mathbf{r}_{i+1} - \mathbf{r}_i\|}, \quad i \in [2, N-1] \tag{3}$$

The internal coordinate system q_i, ϕ_i which we have constructed is shown in S1 Fig. We use these coordinates to define quadratic approximations to the axial and transverse potential energies around the equilibrium state. The axial potential is given by

$$U_a = \frac{1}{2} \sum_{i=1}^{N-1} k_{a,i} q_i^2 \tag{4}$$

where $k_{a,i}$ is the axial stiffness of the i'th body segment. The transverse potential may be written similarly as

$$U_t = \frac{1}{2} \sum_{i=2}^{N-1} k_{t,i} \phi_i^2 \tag{5}$$

where $k_{t,i}$ is the transverse (bending) stiffness about the i'th segment boundary. We account for dissipation of mechanical energy due to viscous friction within the tissues of the larva by approximating axial and transverse power losses by negative definite, quadratic forms in the generalised velocities associated with our internal coordinate system. The axial power loss is then given by

$$P_a = -\sum_{i=1}^{N-1} \eta_{a,i} \dot{q}_i^2 \tag{6}$$

where $\eta_{a,i}$ is the coefficient of viscosity of the i'th body segment. The transverse power loss is

$$P_t = -\sum_{i=2}^{N-1} \eta_{t,i} \dot{\phi}_i^2 \tag{7}$$

where $\eta_{t,i}$ is the coefficient of viscosity associated with bending about the i'th segment boundary. We allow the larva to interact with its substrate via Coulomb kinetic friction, which causes negative definite power losses from the body. During our investigation of small-amplitude dissipative motion, we assume isotropic (direction independent) substrate interaction, with power loss given by

$$P_f = -\sum_{i=1}^{N} \mu_i \|\dot{\mathbf{r}}_i\| \tag{8}$$

where μ_i is a parameter characterising the magnitude of sliding friction forces, and is related to the terrestrial gravitational acceleration g and the coefficient of kinetic friction $\mu_{kinetic}$ of the i'th mass by $\mu_i = \mu_{kinetic} m_i g$. During our investigation of large-amplitude dissipative motion, we allow anisotropic substrate interaction. In this case, the i'th mass is acted upon by a force $F_{friction,i}$ which is directed opposite to its velocity vector v_i and has a magnitude which depends upon the angle θ_i between the velocity vector and the local body axis (see S1 Fig),

$$\mathbf{F}_{friction,i} = -F(\theta_i) \frac{\mathbf{v}_i}{\|\mathbf{v}_i\|} \tag{9}$$

with

$$F(\theta_i) = \mu_{f,i} + (\mu_{b,i} - \mu_{f,i}) \left[\frac{1 - \cos\theta_i}{2} \right]^{\mu_{p,i}} \tag{10}$$

where $\mu_{f,i}$ sets the magnitude of friction opposing motion forward along the body axis ($\theta_i = 0$), $\mu_{b,i}$ sets the magnitude of friction opposing motion backward along the body axis ($\theta_i = \pi$), and $\mu_{p,i} > 0$ sets the directional "focus" of the friction force.

We also allow for flow of power due to muscle activation

$$P_u = -\sum_{i=1}^{N-1} b_i u_i \dot{q}_i \tag{11}$$

where b_i is a (positive) gain parameter, u_i is a dimensionless control variable (identified with muscle activation MF_i in the main text), and the product $b_i u_i$ is the total axial tension across the i'th body segment. As described in the main text, the internal coelomic fluid of the larva gives rise to a constraint on the total length of the larval midline,

$$\sum_{i=1}^{N-1} (l_i + q_i) = L \tag{12}$$

where the summands on the left are the time-dependent lengths of the individual segments of the midline, and L is a constant. Noting that in equilibrium ($q_i = 0$) we must have $\sum_i l_i = L$, we can rewrite the constraint as

$$\sum_{i=1}^{N-1} q_i = 0 \tag{13}$$

It is easy to enforce the total length constraint directly in the case of small amplitude motion or purely axial motion, but in the general case of large amplitude axial and transverse motion this constraint can be difficult to enforce. We therefore attempt to satisfy the constraint only approximately, by introducing an additional potential energy

$$U_c = k_c \left[\sum_{i=1}^{N-1} q_i \right]^2 \tag{14}$$

where the constraint stiffness k_c is chosen to be very large relative to the other stiffness parameters k_a and k_t. Numerically, we satisfy this condition by setting $k_c = \max(k_a, k_t) \times 10^3$.

To derive the dynamics for our system in a form suitable for simulation, we start by using the coordinate transformations 2 and 3 and the definition of the linear momenta $\mathbf{p}_i = m_i \dot{\mathbf{r}}_i$ to write the Hamiltonian function

$$H(\mathbf{r}, \mathbf{p}) = T(\mathbf{p}) + U_a(\mathbf{r}) + U_t(\mathbf{r}) + U_c(\mathbf{r}) \tag{15}$$

which corresponds to the total mechanical energy of the body. We then construct the Rayleigh dissipation function R, which is the sum of the expressions for power transfer into the body, weighted by the inverse homogeneity of each expression [1]. This must also be expressed in terms of the lab frame coordinates and momenta by means of the transformations (2) and (3) and their time derivatives, so that we have

$$R(\mathbf{r}, \mathbf{p}; \mathbf{u}) = \frac{1}{2} P_a(\mathbf{r}, \mathbf{p}) + \frac{1}{2} P_t(\mathbf{r}, \mathbf{p}) + P_f(\mathbf{r}, \mathbf{p}) + P_u(\mathbf{r}, \mathbf{p}; \mathbf{u}) \tag{16}$$

From these two functions, the entire body dynamics can be derived as a system of $2N_{DOF}$ first order differential equations using the dissipative Hamilton's equations

$$\dot{\mathbf{r}}_i = \frac{\partial H}{\partial \mathbf{p}_i} \tag{17}$$

$$\dot{\mathbf{p}}_i = -\frac{\partial H}{\partial \mathbf{r}_i} + \frac{\partial R}{\partial \mathbf{p}_i} \frac{d\mathbf{p}_i}{d\dot{\mathbf{r}}_i} \tag{18}$$

Where our expression for anistropic friction (9) must be added to the right hand side of (18) where appropriate. These differential equations can be solved to find the positions $\mathbf{r}_i$ and momenta $\mathbf{p}_i$ of the masses in the lab frame.

For the sake of brevity we will not write out $H(\mathbf{r}, \mathbf{p})$, $R(\mathbf{r}, \mathbf{p})$, or the dissipative Hamilton's equations in full here. We stress, however, that our model is entirely specified by the expressions for the kinetic and potential energy and the power transfer into the body, along with the transformations between the lab frame and internal coordinates, and the anistropic friction function. We manipulate these expressions in practise using a computer algebra system (`SymPy`). For simulation, we numerically integrate the dissipative Hamilton's equations with pre-specified initial conditions and parameters.

References

1. Landau LD, Lifshitz EM. Mechanics. vol. 1 of Course of Theoretical Physics. 3rd ed. Butterworth-Heinemann; 1976.
