# Supplementary material for: Modelling the mechanics of exploration in larval Drosophila
Source: PLoS Comput Biol. 2019 Jul 5;15(7):e1006635. doi: 10.1371/journal.pcbi.1006635 (PMC6636753; doi:10.1371/journal.pcbi.1006635)
Supplement: S2 Appendix — (PDF) [file pcbi.1006635.s011.pdf]

S2 Appendix – Detailed small-amplitude analysis

In this appendix we present an analysis of the conservative, small oscillations of the larval body. We will attempt to apply the Liouville-Arnold integrability theorem, which tells us that the motion of a mechanical system must be (quasi)periodic if there exist a number of conserved quantities equal to the number of degrees of freedom, and which are in involution (a condition we will define later) [1]. We will find conserved quantities by *separating* the Hamiltonian describing our small-amplitude model. To illustrate this method, suppose we have a Hamiltonian $H(q,p)$ which depends upon a set of generalised coordinates q (not necessarily the axial stretches defined earlier) and their canonically conjugate momenta p, and that this Hamiltonian can be separated into a sum of independent Hamiltonians

$$H(q,p) = H_1(q^1,p^1) + H_2(q^2,p^2) \tag{1}$$

where q^1, q^2 are non-intersecting subsets of q, and p^1, p^2 are the momenta conjugate to these coordinates. Taking the derivative of H_1 and H_2 with respect to time, we find

$$\dot{H}_1 = \frac{\partial H_1}{\partial q^1}\dot{q}^1 + \frac{\partial H_1}{\partial p^1}\dot{p}^1 \tag{2}$$

and

$$\dot{H}_2 = \frac{\partial H_2}{\partial q^2}\dot{q}^2 + \frac{\partial H_2}{\partial p^2}\dot{p}^2 \tag{3}$$

meanwhile, the (conservative) Hamilton's equations tell us that

$$\dot{q}^1 = \frac{\partial H}{\partial p^1} = \frac{\partial H^1}{\partial p^1} \tag{4}$$

$$\dot{q}^2 = \frac{\partial H}{\partial p^2} = \frac{\partial H^2}{\partial p^2} \tag{5}$$

$$\dot{p}^1 = -\frac{\partial H}{\partial q^1} = -\frac{\partial H^1}{\partial q^1} \tag{6}$$

$$\dot{p}^2 = -\frac{\partial H}{\partial q^2} = -\frac{\partial H^2}{\partial q^2} \tag{7}$$

substitution into the expressions above then gives

$$\dot{H}_1 = -\dot{p}^1\dot{q}^1 + \dot{p}^1\dot{q}^1 = 0 \tag{8}$$

$$\dot{H}_2 = -\dot{p}^2\dot{q}^2 + \dot{p}^2\dot{q}^2 = 0 \tag{9}$$

which shows that both H_1 and H_2 are conserved quantities. To test whether these quantities are in involution, we must check that their Poisson bracket vanishes [1,2], i.e. we must check that

$$\{H_1, H_2\} = \sum_k \left(\frac{\partial H_1}{\partial p_k}\frac{\partial H_2}{\partial q_k} - \frac{\partial H_1}{\partial q_k}\frac{\partial H_2}{\partial p_k} \right) = 0 \tag{10}$$

Noting that $q_k \in q^1$ implies $p_k \in p^1$, we see that the partial derivates $\frac{\partial H_2}{\partial q_k}$ and $\frac{\partial H_2}{\partial p_k}$ vanish. Similarly, if $q_k \in q^2$ then $p_k \in p^2$ so that the partial derivatives $\frac{\partial H_1}{\partial q_k}$ and $\frac{\partial H_1}{\partial p_k}$ also vanish. Therefore, every term of the

summation must be equal to zero, so that the Poisson bracket of H_1 and H_2 vanishes, and the quantities are in involution. The above argument can be applied recursively to show that if a Hamiltonian is separable into more than two parts, then those parts are conserved quantities which are in mutual involution.

Let us now begin our investigation of the small-amplitude motions of the larval body. We will do this by taking a Taylor series approximation to the Hamiltonian about the body's equilibrium state, and keeping only terms up to second order. Since the Hamilton's equations give us the dynamics of the larval body by differentiating the Hamiltonian, this second-order approximation is equivalent to linearising the dynamics about the equilibrium.

We first align the midline along the x-axis of the lab frame, with all segment boundaries in their equilibrium positions (i.e. separated by distances l_i along the x-axis). We then construct a new coordinate system such that the variables x_i and y_i denote the displacement of the i'th mass along the x and y axes of the lab frame, respectively, relative to the equilibrium configuration. The canonical momenta $p_{x,i}$, $p_{y,i}$ conjugate to these coordinates are then simply the x and y components of the lab frame momenta $\mathbf{p}_i$. Expanding the Hamiltonian as a Taylor series about the equilibrium $\mathbf{x} = \mathbf{y} = \mathbf{p}_x = \mathbf{p}_y = \mathbf{0}$, and keeping terms up to second order, we obtain the small oscillation Hamiltonian

$$H_{SO}(\mathbf{x}, \mathbf{y}, \mathbf{p}_x, \mathbf{p}_y) = \underbrace{\frac{1}{2}\left[\mathbf{p}_x^T\mathbf{p}_x + \omega_a^2\mathbf{x}^T\mathbf{D}_2\mathbf{x}\right]}_{H_a(\mathbf{x},\mathbf{p}_x)} + \underbrace{\frac{1}{2}\left[\mathbf{p}_y^T\mathbf{p}_y + \omega_t^2\mathbf{y}^T\mathbf{D}_4\mathbf{y}\right]}_{H_t(\mathbf{y},\mathbf{p}_y)} \tag{11}$$

where we have further assumed that all segments of the body are identical, i.e. $m_i = m$, $l_i = l$, $k_{a,i} = k_a$, $k_{t,i} = k_t$, and we have scaled the coordinates $\mathbf{x} \to \mathbf{x}/\sqrt{m}$, $\mathbf{y} \to \mathbf{y}/\sqrt{m}$ to simplify the kinetic energy and absorb all of the mechanical parameters into the potential energy, so that $\omega_a^2 = k_a/m$ and $\omega_t^2 = k_t/ml^2$. $\mathbf{D}_2$ is the $(N-1) \times (N-1)$ circulant second difference matrix

$$\mathbf{D}_2 = \begin{bmatrix} 2 & -1 & & & -1 \\ -1 & 2 & -1 & & \\ & \ddots & \ddots & \ddots & \\ & & -1 & 2 & -1 \\ -1 & & & -1 & 2 \end{bmatrix} \tag{12}$$

and $\mathbf{D}_4$ is the $N \times N$ fourth difference matrix with free boundary conditions

$$\mathbf{D}_4 = \begin{bmatrix} 1 & -2 & 1 & & & & \\ -2 & 5 & -4 & 1 & & & \\ 1 & -4 & 6 & -4 & 1 & & \\ & \ddots & \ddots & \ddots & \ddots & \ddots & \\ & & 1 & -4 & 6 & -4 & 1 \\ & & & 1 & -4 & 5 & -2 \\ & & & & 1 & -2 & 1 \end{bmatrix} \tag{13}$$

For the case of small oscillations the full Hamiltonian is clearly separable into an axial and a transverse Hamiltonian, which we labelled H_a and H_t above. These terms correspond to the total axial and transverse mechanical energy in the larval body, respectively. Our earlier investigation of separable Hamiltonians tells us that each term is an independently conserved quantity, so that no energy transfer may occur between small amplitude axial and transverse motions. Let us now attempt to further separate these Hamiltonians. We will do this by introducing a new set of coordinates $\mathbf{X}, \mathbf{Y}$, called *modal* coordinates, which are linearly related to the axial and transverse coordinates $\mathbf{x}, \mathbf{y}$ by

$$\mathbf{x} = \mathbf{\Phi}_a\mathbf{X}, \quad \mathbf{y} = \mathbf{\Phi}_t\mathbf{Y} \tag{14}$$

The canonical momenta conjugate to $\mathbf{X}, \mathbf{Y}$ are denoted $\mathbf{p}_X, \mathbf{p}_Y$ and are given by the relations $\mathbf{p}_x = \mathbf{\Phi}_a\mathbf{p}_X$ and $\mathbf{p}_y = \mathbf{\Phi}_t\mathbf{p}_Y$. Using these transformations, we may write the axial and transverse Hamiltonians in terms of the modal coordinates and their canonically conjugate momenta as

$$H_a(\mathbf{X}, \mathbf{p}_X) = \frac{1}{2}\left[\mathbf{p}_X^T\mathbf{\Phi}_a^T\mathbf{\Phi}_a\mathbf{p}_X + \omega_a^2\mathbf{X}^T\mathbf{\Phi}_a^T\mathbf{D}_2\mathbf{\Phi}_a\mathbf{X}\right] \tag{15}$$

$$H_t(\mathbf{Y}, \mathbf{p}_Y) = \frac{1}{2}\left[\mathbf{p}_Y^T \mathbf{\Phi}_t^T \mathbf{\Phi}_t \mathbf{p}_Y + \omega_t^2 \mathbf{Y}^T \mathbf{\Phi}_t^T \mathbf{D}_2 \mathbf{\Phi}_t \mathbf{Y}\right] \tag{16}$$

If the coordinate transformations described by $\mathbf{\Phi}_a$ and $\mathbf{\Phi}_t$ are to separate the axial and transverse Hamiltonians into sums of independent terms, we see that the results of the matrix products $\mathbf{\Phi}_a^T \mathbf{\Phi}_a$, $\mathbf{\Phi}_a^T \mathbf{D}_2 \mathbf{\Phi}_a$ and $\mathbf{\Phi}_t^T \mathbf{\Phi}_t$, $\mathbf{\Phi}_t^T \mathbf{D}_4 \mathbf{\Phi}_t$ must be diagonal. We may use this condition to find the form of the transformation matrices $\mathbf{\Phi}_a$ and $\mathbf{\Phi}_t$. To do this, we first note that $\mathbf{D}_2$ and $\mathbf{D}_4$ are real and symmetric, and that each can therefore be factored by eigendecomposition into a product of an orthogonal matrix of eigenvectors and a diagonal matrix of eigenvalues. Therefore, we can write

$$\mathbf{D}_2 = \mathbf{A}\mathbf{\Lambda}_a\mathbf{A}^T, \quad \mathbf{D}_4 = \mathbf{B}\mathbf{\Lambda}_t\mathbf{B}^T \tag{17}$$

where $\mathbf{A}$ is the orthogonal eigenvector matrix and $\mathbf{\Lambda}_a$ the diagonal eigenvalue matrix of $\mathbf{D}_2$. Similarly, $\mathbf{B}$ is the orthogonal eigenvector matrix and $\mathbf{\Lambda}_t$ the diagonal eigenvalue matrix of $\mathbf{D}_4$. We choose to identify the axial coordinate transformation with the axial eigenvector matrix, so that $\mathbf{\Phi}_a = \mathbf{A}$, and identify the transverse coordinate transformation with the transverse eigenvector matrix, so that $\mathbf{\Phi}_t = \mathbf{B}$. By the orthogonality of these matrices, we then have $\mathbf{\Phi}_a^T = \mathbf{\Phi}_a^{-1}$ and $\mathbf{\Phi}_t^T = \mathbf{\Phi}_t^{-1}$. Effecting the eigendecomposition of $\mathbf{D}_2$, the axial Hamiltonian becomes

$$H_a(\mathbf{X}, \mathbf{p}_X) = \frac{1}{2}\left[\mathbf{p}_X^T \mathbf{\Phi}_a^{-1} \mathbf{\Phi}_a \mathbf{p}_X + \omega_a^2 \mathbf{X}^T \mathbf{\Phi}_a^{-1} \mathbf{\Phi}_a \mathbf{\Lambda}_a \mathbf{\Phi}_a^{-1} \mathbf{\Phi}_a \mathbf{X}\right] = \frac{1}{2}\left[\mathbf{p}_X^T \mathbf{p}_X + \omega_a^2 \mathbf{X}^T \mathbf{\Lambda}_a \mathbf{X}\right] \tag{18}$$

or, denoting the i'th eigenvalue of $\mathbf{D}_2$ as $\lambda_{a,i}$,

$$H_a(\mathbf{X}, \mathbf{p}_X) = \sum_{i=1}^{N-1} \frac{1}{2}\left[p_{X,i}^2 + \omega_a^2 \lambda_{a,i} X_i^2\right] \tag{19}$$

Effecting the eigendecomposition of $\mathbf{D}_4$, the transverse Hamiltonian similarly decouples to give

$$H_t(\mathbf{Y}, \mathbf{p}_Y) = \sum_{i=1}^{N} \frac{1}{2}\left[p_{Y,i}^2 + \omega_t^2 \lambda_{t,i} Y_i^2\right] \tag{20}$$

where $\lambda_{t,i}$ denotes the i'th eigenvalue of $\mathbf{D}_4$. These final expressions show that the axial and transverse Hamiltonians are reduced to sums of independent terms, each of which contains just one modal coordinate and its conjugate momentum. Each term corresponds to the total mechanical energy associated with that mode, and is independently conserved according to our earlier results on separable Hamiltonians. This means that no energy transfer can occur between modal coordinates in the case of small oscillations. Given that we now have a number of conserved quantities equal to the number of degrees of freedom of our system, and these quantities are involution with one another, we can invoke the Liouville-Arnold integrability theorem to tell us that our mechanical system must execute periodic or quasiperiodic motion in the case of small oscillations. Indeed, the Hamilton's equations for the i'th modes are

$$\dot{X}_i = \frac{\partial H}{\partial p_{X,i}} = p_{X,i}, \quad \dot{Y}_i = \frac{\partial H}{\partial p_{Y,i}} = p_{Y,i} \tag{21}$$

and

$$\dot{p}_{X,i} = -\frac{\partial H}{\partial X_i} = -\omega_{a,i}^2 X_i, \quad \dot{p}_{Y,i} = -\frac{\partial H}{\partial Y_i} = -\omega_{t,i}^2 Y_i \tag{22}$$

where we have introduced the parameters $\omega_{a,i} = \omega_a\sqrt{\lambda_{a,i}} = (k_a/m)\sqrt{\lambda_{a,i}}$ and $\omega_{t,i} = \omega_t\sqrt{\lambda_{t,i}} = (k_t/ml^2)\sqrt{\lambda_{t,i}}$. These are harmonic oscillator equations in first order form. We can recover the familiar second-order harmonic oscillator equation by differentiating the first equation with respect to time, finding $\dot{p}_{X,i} = \ddot{X}_i$, $\dot{p}_{Y,i} = \ddot{Y}_i$, before substituting into the second equation to give

$$\ddot{X}_i + \omega_{a,i}^2 X_i = 0, \quad \ddot{Y}_i + \omega_{t,i}^2 Y_i = 0, \tag{23}$$

The solution to the harmonic oscillator problem is well known [3], and in this case tells us

$$X_i = A_{a,i}\sin\left(\omega_{a,i}t + \theta_{a,i}\right), \qquad Y_i = A_{t,i}\sin\left(\omega_{t,i}t + \theta_{t,i}\right) \tag{24}$$

These solutions tell us that the modal coordinates execute sinusoidal oscillations with constant amplitude $A_{a,i}$, $A_{t,i}$, phase shift $\theta_{a,i}$, $\theta_{t,i}$, and frequency $\omega_{a,i}$ and $\omega_{t,i}$. To relate this back to our original small oscillation coordinates $\mathbf{x}$ and $\mathbf{y}$ we need to find the eigenvectors $\mathbf{\Phi}_a$, $\mathbf{\Phi}_t$, and the corresponding eigenvalues $\lambda_{a,i}$, $\lambda_{t,i}$, of $\mathbf{D}_2$ and $\mathbf{D}_4$. We can find both $\mathbf{\Phi}_a$ and $\mathbf{\Lambda}_a$ analytically by noting that $\mathbf{D}_2$ is a circulant matrix. Indeed, the i'th eigenvector of an arbitrary circulant matrix is given by [4]

$$\mathbf{\Phi}_{a,i} = \frac{1}{\sqrt{N-1}}\left[1, z_i, z_i^2, \cdots, z_i^{N-2}\right]^T \tag{25}$$

where we have used $\mathbf{\Phi}_{a,i}$ to denote the i'th column of the eigenvector matrix $\mathbf{\Phi}_a$, and $z_i = e^{\frac{2\pi i j}{N-1}}$ is the i'th element of the $(N-1)$'th roots of unity, with $j = \sqrt{-1}$ the imaginary unit. Using Euler's complex exponential formula the k'th element of the i'th axial mode shape may be written

$$\mathbf{\Phi}_{a,k,i} = \frac{1}{\sqrt{N-1}}\left[\cos\left(2\pi i\frac{k}{N-1}\right) + j\sin\left(2\pi i\frac{k}{N-1}\right)\right] \tag{26}$$

The real and complex parts of each vector can be considered as independent mode shapes, so that the modes thus come in pairs with identical spatial frequency,

$$\mathbf{\Phi}_{a,k,i} = \frac{1}{\sqrt{N-1}}\cos\left(2\pi i\frac{k}{N-1}\right), \text{ or } \mathbf{\Phi}_{a,k,i} = \frac{1}{\sqrt{N-1}}\sin\left(2\pi i\frac{k}{N-1}\right), \quad i \in [0, N/2-1] \tag{27}$$

For an arbitrary $(N-1)\times(N-1)$ circulant matrix with entries

$$\mathbf{A} = \begin{bmatrix} c_0 & c_{N-2} & \cdots & c_2 & c_1 \\ c_1 & c_0 & c_{N-2} & & c_2 \\ \vdots & c_1 & c_0 & \ddots & \vdots \\ c_{N-3} & & \ddots & \ddots & c_{N-2} \\ c_{N-2} & c_{N-3} & \cdots & c_1 & c_0 \end{bmatrix} \tag{28}$$

the eigenvalue corresponding to the i'th eigenvector is given by [4]

$$\lambda_i = c_0 + c_{N-2}z_i + c_{N-3}z_i^2 + \cdots + c_1 z_i^{N-2} \tag{29}$$

In the case of $\mathbf{D}_2$ we have $c_0 = 2$ and $c_1 = c_{N-2} = -1$, so that this reduces to

$$\lambda_i = 2 - z_i - z_i^{N-2} \tag{30}$$

However, the $N-1$'th roots of unity satisfy $z_i^{N-2} = \bar{z}_i$, where the bar indicates the complex conjugate. Therefore,

$$\lambda_i = 2 - 2\mathrm{Re}[z_i] \tag{31}$$

the real part of z_i can be found by using Euler's complex exponential formula, yielding

$$\lambda_i = 2 - 2\cos\left(\frac{2\pi i}{N-1}\right) \tag{32}$$

By using the trigonometric identity $\sqrt{2-2\cos(x)} = 2\sin\left(\frac{x}{2}\right)$ we may now calculate the frequency of oscillation of the i'th axial mode to be

$$\omega_{a,i} = 2\omega_a\left|\sin\left(\frac{\pi i}{N-1}\right)\right| \tag{33}$$

It is marked that the axial modes come in pairs with identical temporal and spatial frequencies. This property allows us to construct travelling wave solutions for the axial motion by combining sinusoidal

oscillations within a pair of modes with equal magnitude and a $90°$ temporal phase shift relative to each other. To see this mathematically, we re-examine our expression for the axial mode shapes (27). We multiply these vectors by modal coordinates oscillating with unity amplitude, identical temporal frequency $\omega_{a,i}$ and a $\pm 90°$ phase shift, and sum the result, so that the k'th segment boundary displacement for the i'th axial mode can be written

$$x_k = \cos\left(\omega_{a,i}t\right)\cos\left(2\pi i\frac{k}{N-1}\right) + \cos\left(\omega_{a,i}t \pm \frac{\pi}{2}\right)\sin\left(2\pi i\frac{k}{N-1}\right) \tag{34}$$

where we have dropped the normalising factor $\frac{1}{\sqrt{N-1}}$ in (27). This is equivalent to

$$x_k = \cos\left(\omega_{a,i}t\right)\cos\left(2\pi i\frac{k}{N-1}\right) \pm \sin\left(\omega_{a,i}t\right)\sin\left(2\pi i\frac{k}{N-1}\right) \tag{35}$$

Using the identity $\cos(a)\cos(b) \pm \sin(a)\sin(b) = \cos(a \pm b)$, this further simplifies to

$$x_k = \cos\left(\omega_{a,i}t \pm 2\pi i\frac{k}{N-1}\right) \tag{36}$$

Interpreting $0 \leq \frac{k}{N-1} \leq 1$ as a spatial coordinate ranging over the undeformed configuration of the body, this is in the form of a sinusoidal travelling wave, and the choice of a minus or plus sign in the argument corresponds to the choice of a forward- or backward-propagating wave, respectively.

References

1. Arnol'd VI. Mathematical Methods of Classical Mechanics. vol. 60 of Graduate Texts in Mathematics. 2nd ed. Springer; 1989.

2. Landau LD, Lifshitz EM. Mechanics. vol. 1 of Course of Theoretical Physics. 3rd ed. Butterworth-Heinemann; 1976.

3. Tenenbaum M, Pollard H. Ordinary Differential Equations. 1st ed. Dover Books on Mathematics. Dover Publications, Inc.; 1985.

4. Gray RM. Toeplitz and Circulant Matrices: A review. 1st ed. Now Publishers Inc.; 2006.
