# Supplementary material for: Modelling the mechanics of exploration in larval Drosophila
Source: PLoS Comput Biol. 2019 Jul 5;15(7):e1006635. doi: 10.1371/journal.pcbi.1006635 (PMC6636753; doi:10.1371/journal.pcbi.1006635)
Supplement: S3 Appendix — (PDF) [file pcbi.1006635.s012.pdf]

S3 Appendix – A trade-off between power flow into the body and force on the centre of mass

The neural model we have constructed in this paper was motivated by the requirement for power flow from the musculature to the body, and by the requirement that a small number of segments should move in the direction of centre of mass motion at any given time. There is in fact an inherent trade-off between the need to transfer power into the body and the need to move only a small number of segments, as we will now show by extending the modal analysis of the previous appendix to the dissipative axial mechanics. The Hamiltonian and Rayleigh dissipation function in this case reduce to

$$H_a(\mathbf{x}, \mathbf{p}_x) = \frac{1}{2}\left[\mathbf{p}_x^T \mathbf{p}_x + \omega_a^2 \mathbf{x}^T \mathbf{D}_2 \mathbf{x}\right] \tag{1}$$

$$R_a(\mathbf{x}, \mathbf{p}_x) = -\zeta_a \omega_a \mathbf{p}_x^T \mathbf{D}_2 \mathbf{p}_x + b\mathbf{p}_x^T \mathbf{D}_1 \mathbf{u} \tag{2}$$

where we have again assumed that all segments have identical parameters and we have neglected sliding friction for simplicity, and we have defined the axial damping ratio $\zeta_a = \eta_a / 2\sqrt{k_a m}$. $\mathbf{D}_1$ is the circulant first difference matrix

$$D_1 = \begin{bmatrix} 1 & & & -1 \\ -1 & 1 & & \\ & \ddots & \ddots & \\ & & -1 & 1 \end{bmatrix} \tag{3}$$

We now move to the axial modal basis $\mathbf{X}$, $\mathbf{p}_X$ defined in the previous appendix by $\mathbf{x} = \mathbf{\Phi}_a \mathbf{X}$, $\mathbf{p}_x = \mathbf{\Phi}_a \mathbf{p}_X$, and introduce modal control variables $\mathbf{U}$ defined by $\mathbf{u} = \mathbf{\Phi}_a \mathbf{U}$, finding

$$H_a(\mathbf{X}, \mathbf{p}_X) = \frac{1}{2}\left[\mathbf{p}_X^T \mathbf{p}_X + \omega_a^2 \mathbf{X}^T \mathbf{\Lambda}_a \mathbf{X}\right] \tag{4}$$

$$R_a(\mathbf{X}, \mathbf{p}_X) = -\zeta_a \omega_a \mathbf{p}_X^T \mathbf{\Lambda}_a \mathbf{p}_X + b\mathbf{p}_X^T \mathbf{\Sigma} \mathbf{U} \tag{5}$$

where we have used the fact that the matrix $\mathbf{D}_1$ is circulant and is therefore diagonalised by the eigenvector matrix $\mathbf{\Phi}_a$ of $\mathbf{D}_2$, since all circulant matrices have the same eigenvectors. We write the diagonal eigenvalue matrix of $\mathbf{D}_1$ as $\mathbf{\Sigma}$, with the i'th eigenvalue being $\mathbf{\Sigma}_{i,i} = \sigma_i$. The dissipative Hamilton's equations for the i'th axial mode are then

$$\dot{X}_i = \frac{\partial H_a}{\partial p_{X,i}} = p_{X,i} \tag{6}$$

$$\dot{p}_{X,i} = -\frac{\partial H_a}{\partial X_i} + \frac{\partial R_a}{\partial p_{X,i}} \frac{dp_{X,i}}{d\dot{X}_i} = -\omega_a^2 \lambda_{a,i} X_i - 2\zeta_a \omega_a \lambda_{a,i} p_{X,i} + b\sigma_i U_i \tag{7}$$

Where we have used the first equation to tell us that $\frac{d\mathbf{p}_X}{d\dot{\mathbf{X}}} = 1$ in the second equation. We now convert this system of first-order equations to a single second order equation by using the first equation to write $\dot{X}_i = p_{X,i}$ and $\ddot{X}_i = \dot{p}_{X,i}$ in the second, finding

$$\ddot{X}_i + 2\zeta_a \omega_a \lambda_{a,i} \dot{X}_i + \omega_a^2 \lambda_{a,i} X_i = b\sigma_i U_i \tag{8}$$

Finally, if we introduce the modal frequencies as $\omega_{a,i} = \omega_a \sqrt{\lambda_{a,i}}$, the modal damping ratios as $\zeta_{a,i} = \zeta_a \sqrt{\lambda_{a,i}}$, and the modal gain factors as $b_i = b\sigma_i$, we obtain the equation of motion for a damped, driven, harmonic oscillator in standard form

$$\ddot{X}_i + 2\zeta_{a,i}\omega_{a,i}\dot{X}_i + \omega_{a,i}^2 X_i = b_i U_i \tag{9}$$

The ratio of energy stored to energy dissipated per cycle of oscillation for a damped harmonic oscillator is given by the Q-factor, defined as $Q = 1/2\zeta$. The lower frequency modes, corresponding to small eigenvalues $\lambda_{a,i}$, will have lower damping ratios $\zeta_{a,i}$ and therefore higher Q-factors. In other words, energy is more efficiently transferred into the low-frequency modes. However, solely driving the lowest frequency modes would fail to produce any force on the centre of mass, because the resulting peristaltic wave would involve equal numbers of segments moving forward and backward, and the resulting frictive forces would cancel out. This necessitates some involvement of higher-frequency modes in order to localise segmental motion and allow overall acceleration of the body. Thus, there is a trade-off between efficiently transferring power into low-frequency modes and producing large forces on the centre of mass.
