# Supplementary material for: Modelling the mechanics of exploration in larval Drosophila
Source: PLoS Comput Biol. 2019 Jul 5;15(7):e1006635. doi: 10.1371/journal.pcbi.1006635 (PMC6636753; doi:10.1371/journal.pcbi.1006635)
Supplement: S4 Appendix — (PDF) [file pcbi.1006635.s013.pdf]

S4 Appendix – Modelling and analysis of head motion

In this appendix we will consider bending and compression/expansion of the head segment, as viewed from a coordinate frame fixed at the posterior end of the head segment and aligned with the local body axis (the head frame). To simplify our notation we will drop indices, denoting the head's bending angle as $\phi = \phi_{N-1}$ and the head's stretch as $q = q_{N-1}$. The potential energy of the head segment in this frame of reference is then simply

$$U_h = \frac{1}{2}k_a q^2 + \frac{1}{2}k_t \phi^2 \quad (1)$$

Meanwhile, the kinetic energy takes the form

$$T_h = \frac{1}{2m}p_q^2 + \frac{1}{2m(l+q)^2}p_\phi^2 + m(l+q)\left[W_x cos(\phi) + W_y sin(\phi)\right] - \Omega p_\phi \quad (2)$$

where W_x, W_y give the translational acceleration, and Ω the angular velocity, of the head frame relative to the lab frame. The first two terms in this expression can be seen as the kinetic energy obtained by treating the head frame as an inertial reference frame, while the second two terms involving W_x, W_y, and Ω are a correction accounting for non-inertial effects (i.e. fictitious forces) arising in the head frame. Aiming to simplify our analysis as far as possible, we choose to neglect the non-inertial effects, so that the Hamiltonian of the system becomes

$$H_h = \frac{1}{2m}p_q^2 + \frac{1}{2m(l+q)^2}p_\phi^2 + \frac{1}{2}k_a q^2 + \frac{1}{2}k_t \phi^2 \quad (3)$$

We can simplify this expression further by choosing to measure length, mass, and time in convenient units, such that $m = 1$, $l = 1$, and $\omega_a = \sqrt{k_a/m} = 1$. In this case, the Hamiltonian becomes

$$H_h = \frac{1}{2}\left[p_q^2 + \frac{1}{(1+q)^2}p_\phi^2 + q^2 + \lambda^2\phi^2\right] \quad (4)$$

where $\lambda = \omega_t/\omega_a$ is the ratio of transverse to axial frequencies. We can also introduce an explicit amplitude scale by multiplying all dynamical variables by a parameter ϵ. The head Hamiltonian then becomes

$$H_h = \frac{1}{2}\left[\epsilon^2 p_q^2 + \frac{1}{(1+\epsilon q)^2}\epsilon^2 p_\phi^2 + \epsilon^2 q^2 + \epsilon^2\lambda^2\phi^2\right] \quad (5)$$

or, dividing through by ϵ^2,

$$H_h^* = \frac{H_h}{\epsilon^2} = \frac{1}{2}\left[p_q^2 + \frac{1}{(1+\epsilon q)^2}p_\phi^2 + q^2 + \lambda^2\phi^2\right] \quad (6)$$

We can see immediately that for the case of small oscillations, i.e. $\epsilon \to 0$, the head Hamiltonian reduces to the simpler expression

$$H_{h,SO}^* = \frac{1}{2}\left[p_q^2 + q^2\right] + \frac{1}{2}\left[p_\phi^2 + \lambda^2\phi^2\right] \quad (7)$$

which is clearly separable into an axial and a transverse Hamiltonian. By our investigation of separable Hamiltonians (S2 Appendix) theorems we know that these are both conserved quantities, and that they are in involution with one another. Furthermore, this tells us that the motion of the head has a closed-form solution and must be (quasi)periodic. Indeed, the Hamilton's equations in this case tell us

$$\dot{q} = \frac{\partial H^*_{h,SO}}{\partial p_q} = p_q, \quad \dot{\phi} = \frac{\partial H^*_{h,SO}}{\partial p_\phi} = p_\phi \tag{8}$$

and

$$\dot{p}_q = -\frac{\partial H^*_{h,SO}}{\partial q} = -q, \quad \dot{p}_\phi = -\frac{\partial H^*_{h,SO}}{\partial \phi} = -\lambda^2 \phi \tag{9}$$

which are harmonic oscillator equations in first order form. As before (S2 Appendix), we can recover the familiar second-order harmonic oscillator equation by differentiating the first equations with respect to time, finding $\dot{p}_q = \ddot{q}$, $\dot{p}_\phi = \ddot{\phi}$, before substituting this result into the second equations to find

$$\ddot{q} + q = 0, \quad \ddot{\phi} + \lambda^2 \phi = 0 \tag{10}$$

The solution to the harmonic oscillator problem is well known [1], and in this case tells us

$$q(t) = A_q \cos(t + \theta_q), \quad \phi(t) = A_\phi \cos(\lambda t + \theta_\phi) \tag{11}$$

where the A's are constant amplitudes and the θ's are constant phase shifts. It should be clear from this solution that if λ is rational, the head motion will be periodic while for irrational λ the motion will be quasiperiodic.

To gain insight into the more general case of large amplitude head motion, we attempt to apply the Kolmogorov-Arnold-Moser (KAM) theorem, a key result in classical mechanics [2]. The KAM theorem tells us that for sufficiently small conservative perturbations of an integrable Hamiltonian the motion remains quasiperiodic for a majority of initial conditions, while the region of phase space occupied by chaotic behaviour increases in size with the magnitude of perturbation. The KAM theorem first requires that we write the head Hamiltonian as a sum of an integrable unperturbed Hamiltonian H_0 and a small conservative perturbation ϵH_1, i.e.

$$H^*_h = H_0 + \epsilon H_1 \tag{12}$$

There are in principle several ways of accomplishing this. We proceed by taking a Taylor series expansion in ϵ,

$$H^*_h = H_0 + \epsilon \frac{\partial H^*_h}{\partial \epsilon} + \frac{\epsilon^2}{2} \frac{\partial^2 H^*_h}{\partial \epsilon^2} + \cdots = H_0 + \epsilon \underbrace{\left[\frac{\partial H^*_h}{\partial \epsilon} + \frac{\epsilon}{2} \frac{\partial^2 H^*_h}{\partial \epsilon^2} + \cdots \right]}_{H_1} \tag{13}$$

where we have identified H_0 with the zero'th order term in the expansion, which is simply the small oscillation head Hamiltonian $H_0 = H^*_h(\epsilon = 0) = H^*_{h,SO}$, which we know to be integrable. Unfortunately, this means that the unperturbed system is governed by a harmonic oscillator Hamiltonian (see above), which fails to meet the isoenergetic nondegeneracy condition of the KAM theorem [2]. We therefore cannot formally apply the KAM theorem to the problem of head motion. Nevertheless, numerical experiments do suggest that quasiperiodic behaviour persists for small perturbations, while larger perturbations imply that a greater region of phase space will be occupied by chaos (see main text).

References

1. Tenenbaum M, Pollard H. Ordinary Differential Equations. 1st ed. Dover Books on Mathematics. Dover Publications, Inc.; 1985.

2. Arnol'd VI. Mathematical Methods of Classical Mechanics. vol. 60 of Graduate Texts in Mathematics. 2nd ed. Springer; 1989.
