# Supplementary material for: Modelling the mechanics of exploration in larval Drosophila
Source: PLoS Comput Biol. 2019 Jul 5;15(7):e1006635. doi: 10.1371/journal.pcbi.1006635 (PMC6636753; doi:10.1371/journal.pcbi.1006635)
Supplement: S5 Appendix — (PDF) [file pcbi.1006635.s014.pdf]

S5 Appendix – Effective body physics arising due to relationship of neuromuscular action to body motion

In this appendix we describe how the action of the neuromuscular system can effectively alter physical properties of the body. Consider an arbitrary segment of the larval body, whose kinematics are described by an axial stretch q and a transverse bending angle ϕ. This segment's contributions to the elastic potential energy and Rayleigh dissipation function are

$$U = \frac{1}{2}k_a q^2 + \frac{1}{2}k_t \phi^2 \tag{1}$$

$$R = -\frac{1}{2}\eta_a \dot{q}^2 - \frac{1}{2}\eta_t \dot{\phi}^2 \tag{2}$$

Suppose the nervous system activates the local musculature so as to exert upon the segment an axial tension u_a and a bending torque u_t, both having a fixed relationship to the local mechanical state, so that we may write $u_a = u_a(q, \dot{q})$, $u_t = u_t(\phi, \dot{\phi})$. This could correspond to the action of local reflex arcs or to the action of a central pattern generator which has been entrained to the body's motion. The total generalised axial and transverse forces Q_a, Q_t due to viscoelastic body mechanics and muscular activation are then given by

$$Q_a = -\frac{\partial U}{\partial q} + \frac{\partial R}{\partial \dot{q}} + u_a(q, \dot{q}) = -k_a q - \eta_a \dot{q} + u_a(q, \dot{q}) \tag{3}$$

$$Q_t = -\frac{\partial U}{\partial \phi} + \frac{\partial R}{\partial \dot{\phi}} + u_t(\phi, \dot{\phi}) = -k_t \phi - \eta_t \dot{\phi} + u_t(\phi, \dot{\phi}) \tag{4}$$

Expanding the muscular terms as Taylor series up to first order gives

$$u_a(q, \dot{q}) \approx u_a(0,0) + \frac{\partial u_a}{\partial q}(0,0) q + \frac{\partial u_a}{\partial \dot{q}}(0,0)\dot{q} \tag{5}$$

$$u_t(\phi, \dot{\phi}) \approx u_t(0,0) + \frac{\partial u_t}{\partial \phi}(0,0)\phi + \frac{\partial u_t}{\partial \dot{\phi}}(0,0)\dot{\phi} \tag{6}$$

We neglect the leading constant terms, since these correspond only to shifts of the equilibrium stretch and bending angle. Keeping only the linear terms, and labelling the coefficients by α and β as shown, the generalised forces become

$$Q_a = -(k_a - \alpha_a) q - (\eta_a - \beta_a)\dot{q} \tag{7}$$

$$Q_t = -(k_t - \alpha_t)\phi - (\eta_t - \beta_t)\dot{\phi} \tag{8}$$

From which it should be clear that stretch- or bend-dependent muscle activation gives an effective shift in the axial or transverse stiffness (and therefore also the natural frequencies of axial and transverse motion, see earlier appendices), while stretch rate- or bend rate-dependent muscle activation gives an effective shift in the axial or transverse coefficient of viscosity.
