# Supplementary material for: Modelling the mechanics of exploration in larval Drosophila
Source: PLoS Comput Biol. 2019 Jul 5;15(7):e1006635. doi: 10.1371/journal.pcbi.1006635 (PMC6636753; doi:10.1371/journal.pcbi.1006635)
Supplement: S6 Appendix — (PDF) [file pcbi.1006635.s015.pdf]

S6 Appendix – Computer algebra and numerical methods

In preparing this paper we have made use of the `SymPy` computer algebra package for Python. In particular, we used `SymPy` to convert our mathematical expressions for kinetic and potential energies, power losses, and friction forces into a consistent cartesian coordinate system (the lab frame), before combining them to form expressions for the Hamiltonian and Rayleigh dissipation function (see S1 Appendix for definitions of these quantitites). We then used `SymPy` to derive our mechanical equations of motion using the dissipative Hamiltons equations (see S1 Appendix for definitions of these equations).

We carried out our modal analysis using a combination of manual methods and `SymPy`, and used the `NumPy` function `numpy.linalg.eig` to numerically estimate the eigenvalues and eigenvectors of $\mathbf{D}_4$.

We also formulated our neuromuscular model in `SymPy`. In order to avoid algebraic inconsistencies associated with the mutual inhibitory connections in our model, we used a fast switching dynamics. We replaced the algrebaic binary activation function (Eq 4, main text) with the first-order dynamics

$$\dot{V}_i = \begin{cases} r - rV_i & \sum_j w_j V_j > \theta_i \\ -rV_i & \text{otherwise} \end{cases} \tag{1}$$

where r is a switching rate constant. We set $r = 1\ \text{ms}^{-1}$, so that neural state changes occured on a far faster timescale than the evolution of the mechanics.

Having assembled the dynamical equations for our model in `SymPy`, we then used the `sympy.fcode` function to generate equivalent `FORTRAN` source code, and called the `odespy` package to compile this source code and numerically integrate our model equations via the `LSODES` solver. We chose timesteps and absolute/relative tolerances for `LSODES` by requiring that integration of our conservative equations lead to a change in total energy of less than 0.1%. Following simulation, we scaled the time axis so that the fundamental frequency of tail segment length oscillations was 1 Hz, to roughly match observations of the real larva. Note that this is equivalent to scaling the axial and transverse natural frequencies of our model, via stiffness or mass parameters (see S2 Appendix). After scaling, we downsampled to a 30 Hz sampling rate to match typical video recording apparatus.

Given the time series of a quantity x obtained from a simulation run of duration T with timestep Δt, we computed the power spectral density of x as

$$\mathcal{S}\left[x\right](\omega) = \frac{\Delta t^2}{T} \left|\mathcal{F}\left[x\right](\omega)\right|^2 \tag{2}$$

where ω is frequency and F is the fast Fourier transform of x, obtained via the `scipy.fftpack.fft` function in `SciPy`. We computed the autocorrelation of x as

$$\mathcal{A}\left[x\right](t) = x(t) * x(-t) \tag{3}$$

where the star denotes convolution, which we achieved via the `scipy.fftpack.fftconvolve` function. At several points in the paper we were required to fit data by known distributions or curves. We performed linear least-squares fitting of mean-squared displacement (Fig 10, S5 Fig) and log angular speed (S4 Fig) using the `sympy.stats.linregress` function. We computed maximum-likelihood von Mises and wrapped Cauchy fits to the body bend distribution (Fig 10) using `sp.stats.vonmises.fit` and `sp.stats.wrapcauchy.fit`, respectively. We compared exponential and power law fits to the run length distribution (Fig 10) using the `powerlaw` Python package.

To examine the putative chaotic behaviour of our model we calculated the maximal Lyapunov characteristic exponent (MLCE). The MLCE is defined as

$$\lambda = \lim_{t\to\infty} \frac{1}{t} ln \frac{\|\delta x(t)\|}{\|\delta x(0)\|} \tag{4}$$

where $\delta x(0)$ is some initial separation distance between two mechanical trajectories of the system and $\delta x(t)$ is the separation distance at a time t in the future. Technically, limit sets of an N-dimensional system will have a spectrum of N Lyapunov exponents, but the dynamics on the set will be dominated by the largest (maximal) exponent.

We estimated the MLCE using a standard "pullback" algorithm in which two trajectories of the system are numerically integrated in parallel while periodically resetting one to be a distance d_0 away from the other. This procedure can be described as follows :

1. choose some initial conditions for the model and numerically integrate until transient behaviour has diminished; call the final state x_0
2. pick a random vector y_0 which is a distance $\delta x(0)$ from x_0
3. numerically integrate the model dynamics starting from x_0 and y_0 over some time t until reaching states x_1 and y_1
4. calculate the distance between x_1 and y_1 using any vector norm (we use the standard Euclidean norm); denote this distance $\delta x(t)$
5. compute and store a finite time estimate of the MLCE by substituting into the definition above
6. reset $x_0 \to x_1$ and $y_0 \to x_1 + \delta x(0)\frac{(y_1 - x_1)}{\|y_1 - x_1\|}$; i.e. "pull back" y_1 along the vector from x_1 to y_1 until a distance $\delta x(0)$ from x_1
7. repeat steps 3–6 for some fixed number of iterations
8. average over the stored finite time estimates of the MLCE to obtain a single MLCE estimate; some of the initial stored estimates may be discarded first to ensure the y trajectory has aligned along the direction with the largest Lyapunov exponent
