# Supplementary material for: Modelling the mechanics of exploration in larval Drosophila
Source: PLoS Comput Biol. 2019 Jul 5;15(7):e1006635. doi: 10.1371/journal.pcbi.1006635 (PMC6636753; doi:10.1371/journal.pcbi.1006635)
Supplement: S1 Table — All segments are identical. Values given in larval units (seg = resting segment length, segmass = mass of a single segment boundary, nondim = dimensionless/nondimensional). (PDF) [file pcbi.1006635.s016.pdf]

Table S1: neural parameter values. All segments are identical. Values given in larval units (seg = resting segment length, segmass = mass of a single segment boundary, nondim = dimensionless/nondimensional).

| symbol | description | value |
|---|---|---|
| $\theta_{\mathrm{SN}}$ | sensory neuron threshold | 1/100 seg $s^{-1}$ |
| $\theta_{\mathrm{IN}}$ | interneuron threshold | 1/2 (nondim.) |
| $\theta_{\mathrm{MN}}$ | motor neuron threshold | 1/2 (nondim.) |
| $w_{\mathrm{SN}\to\mathrm{MN}}$ | sensory neuron → motor neuron synaptic weight | 1 (nondim.) |
| $w_{\mathrm{MN}\to\mathrm{MF}}$ | motor neuron → muscle fibre synaptic weight | 1 (nondim.) |
| $w_{\mathrm{SN}\to\mathrm{IN}}$ | sensory neuron → interneuron synaptic weight | 1 (nondim.) |
| $w_{\mathrm{IN}\to\mathrm{MN}}$ | interneuron → motor neuron synaptic weight | −2 (nondim.) |
| $w_{\mathrm{IN}\to\mathrm{IN}}$ | interneuron → interneuron synaptic weight | −2 (nondim.) |
