# Supplementary material for: Modelling the mechanics of exploration in larval Drosophila
Source: PLoS Comput Biol. 2019 Jul 5;15(7):e1006635. doi: 10.1371/journal.pcbi.1006635 (PMC6636753; doi:10.1371/journal.pcbi.1006635)
Supplement: S2 Table — All segments are identical. Values given in larval units (seg = resting segment length, segmass = mass of a single segment boundary, nondim = dimensionless/nondimensional). (PDF) [file pcbi.1006635.s017.pdf]

Table S2: mechanical parameters for **Fig 4. Emergence of limit cycles for forward and backward locomotion in the dissipative, small-amplitude model**. All segments are identical. Values given in larval units (seg = resting segment length, segmass = mass of a single segment boundary, nondim = dimensionless/nondimensional).

symbol	description	value
b	reflex gain	varies (see figure) segmass seg s^{-2}
l	equilibrium segment length	1 seg
m	segment mass	1 segmass
k_a	axial stiffness	$(2\pi)^2$ segmass s^{-2}
η_a	axial coefficient of viscosity	2π segmass s^{-1}
μ	coefficient of friction	1 segmass seg s^{-2}
