# Supplementary material for: Modelling the mechanics of exploration in larval Drosophila
Source: PLoS Comput Biol. 2019 Jul 5;15(7):e1006635. doi: 10.1371/journal.pcbi.1006635 (PMC6636753; doi:10.1371/journal.pcbi.1006635)
Supplement: S3 Table — Values given in larval units (seg = resting segment length, segmass = mass of a single segment boundary, nondim = dimensionless/nondimensional). (PDF) [file pcbi.1006635.s018.pdf]

Table S3: mechanical parameters for **Fig 6** and **Fig 7. Emergence of deterministic chaos in the conservative head dynamics as amplitude of motion is increased**. Values given in larval units (seg = resting segment length, segmass = mass of a single segment boundary, nondim = dimensionless/nondimensional).

symbol	description	value
ϵ	amplitude	varies (see figure) (nondim.)
λ	ratio of transverse and axial frequencies	$e/6$ (nondim.)
E	total mechanical energy	$1/2$ (nondim.)
