# Supplementary material for: Modelling the mechanics of exploration in larval Drosophila
Source: PLoS Comput Biol. 2019 Jul 5;15(7):e1006635. doi: 10.1371/journal.pcbi.1006635 (PMC6636753; doi:10.1371/journal.pcbi.1006635)
Supplement: S4 Table — All segments are identical. Values given in larval units (seg = resting segment length, segmass = mass of a single segment boundary, nondim = dimensionless/nondimensional). (PDF) [file pcbi.1006635.s019.pdf]

Table S4: mechanical parameters for **Fig 8. Conservative planar motion of the body is chaotic at large amplitudes**. All segments are identical. Values given in larval units (seg = resting segment length, segmass = mass of a single segment boundary, nondim = dimensionless/nondimensional).

symbol	description	value
l	equilibrium segment length	1 seg
m	segment mass	1 segmass
k_a	axial stiffness	3.15 segmass s^{-2}
k_t	transverse stiffness	8.45 segmass seg^2 s^{-2} rad^{-1}
k_c	incompressibility constraint stiffness	1000 segmass s^{-2}
