# Supplementary material for: Modelling the mechanics of exploration in larval Drosophila
Source: PLoS Comput Biol. 2019 Jul 5;15(7):e1006635. doi: 10.1371/journal.pcbi.1006635 (PMC6636753; doi:10.1371/journal.pcbi.1006635)
Supplement: S5 Table — Values given in larval units (seg = resting segment length, segmass = mass of a single segment boundary, nondim = dimensionless/nondimensional). (PDF) [file pcbi.1006635.s020.pdf]

Table S5: mechanical parameters for **Fig 9. Dissipative planar motion is chaotic** and **Fig 10. Deterministic exploration**. Values given in larval units (seg = resting segment length, segmass = mass of a single segment boundary, nondim = dimensionless/nondimensional)

symbol	description	value
l	equilibrium segment length	1 seg
m	segment mass	1 segmass
k_a	axial stiffness	2 segmass s^{-2}
η_a	axial coefficient of viscosity	0.25 segmass s^{-1}
$k_{t,11}$	transverse stiffness about T1-T2 boundary	0.05 segmass seg^2 s^{-2} rad^{-1}
$k_{t,10}$	transverse stiffness about T2-T3 boundary	0.07 segmass seg^2 s^{-2} rad^{-1}
$k_{t,9}$	transverse stiffness about T3-A1 boundary	0.1 segmass seg^2 s^{-2} rad^{-1}
$k_{t,8}$	transverse stiffness about A1-A2 boundary	0.15 segmass seg^2 s^{-2} rad^{-1}
$k_{t,2}, k_{t,3}, \ldots, k_{t,7}$	transverse stiffness about A7-A8, A6-A7, ..., A2-A3 boundaries	0.2 segmass seg^2 s^{-2} rad^{-1}
$\eta_{t,11}$	transverse viscosity about T1-T2 boundary	0.033 segmass seg^2 s^{-1} rad^{-1}
$\eta_{t,10}$	transverse viscosity about T2-T3 boundary	0.067 segmass seg^2 s^{-1} rad^{-1}
$\eta_{t,9}$	transverse viscosity about T3-A1 boundary	0.33 segmass seg^2 s^{-1} rad^{-1}
$\eta_{t,8}$	transverse viscosity about A1-A2 boundary	0.67 segmass seg^2 s^{-1} rad^{-1}
$\eta_{t,2}, \eta_{t,3}, ldots, \eta_{t,7}$	transverse viscosity about A7-A8, A6-A7, ..., A2-A3 boundaries	1.67 segmass seg^2 s^{-1} rad^{-1}
$\mu_{f,1}, \mu_{f,2}, \ldots, \mu_{f,12}$	(forward) coefficient of friction, all segments	0 segmass seg s^{-2}
$\mu_{b,12}$	(backward) coefficient of friction at head extremity	100 segmass seg s^{-2}
$\mu_{b,11}$	(backward) coefficient of friction at T1-T2 boundary	100 segmass seg s^{-2}
$\mu_{b,10}$	(backward) coefficient of friction at T2-T3 boundary	0.2 segmass seg s^{-2}
$\mu_{b,9}$	(backward) coefficient of friction at T3-A1 boundary	0.2 segmass seg s^{-2}
$\mu_{b,1}, \mu_{b,2}, \ldots, \mu_{b,8}$	(backward) coefficient of friction at tail extremity and A7-A8, ..., A1-A2 boundaries	5 segmass seg s^{-2}
$\mu_{p,12}$	directional "focus" of friction at head extremity	4.5 (nondim.)
$\mu_{p,11}$	directional "focus" of friction at T1-T2 boundary	4.5 (nondim.)
$\mu_{p,1}, \mu_{p,2}, \ldots, \mu_{p,10}$	directional "focus" of friction at tail extremity and A7-A8, ..., T2-T3 boundaries	1.5 (nondim.)
b_9, b_{10}, b_{11}	reflex gain in segments T3, T2, T1	0.09 segmass seg s^{-2}
$b_1, b_2, \ldots, b_8$	reflex gain in segments A8, A7, ..., A1	0.48 N
k_c	incompressibility constraint stiffness	1000 segmass s^{-2}
